# Knowledge and Attitudes around First Aid and Basic Life Support of Kindergarten and Elementary School Teachers and Parents in Taif City, Saudi Arabia

**DOI:** 10.3390/children10071266

**Published:** 2023-07-22

**Authors:** Shadi Tamur, Raghad Mousa Alasmari, Matooqa Abdulkareem Alnemari, Manar Abdullah Altowairgi, Atheer Hammad Altowairqi, Nouf Mohammed Alshamrani, Maryam Aljaid, Sultan Al-Malki, Abdullah Khayat, Ahmad Alzahrani, Anwar Shams

**Affiliations:** 1 Department of Pediatrics, College of Medicine, Taif University, P.O. Box 11099, Taif 21944, Saudi Arabia; shaditamur@tu.edu.sa (S.T.); maryam@tu.edu.sa (M.A.); sultanab@tu.edu.sa (S.A.-M.); khayatam@tu.edu.sa (A.K.); as.alzahrani@tu.edu.sa (A.A.); 2 Faculty of Medicine, Taif University, P.O. Box 11099, Taif 21944, Saudi Arabia; raghadalasmary@gmail.com (R.M.A.); matooqa.19@gmail.com (M.A.A.); manarabdullah.tw@gmail.com (M.A.A.); atheerh.altowairqi@gmail.com (A.H.A.); drnouf.18@gmail.com (N.M.A.); 3 Department of Pharmacology, College of Medicine, Taif University, P.O. Box 11099, Taif 21944, Saudi Arabia; 4 Centre of Biomedical Sciences Research (CBSR), Taif University, P.O. Box 11099, Taif 21974, Saudi Arabia; 5 High Altitude Research Center, Taif University, P.O. Box 11099, Taif 21944, Saudi Arabia

**Keywords:** basic life support, first aid, cardiopulmonary resuscitation, kindergarten teachers, elementary teachers, parents, knowledge, attitude

## Abstract

Background: The foremost cause of children’s (1–19 year) death is inadvertent injuries. While most of these accidental harms occur at home and school, rapid and suitable parental and teacher intervention is required to increase the chances of a child’s survival. Therefore, both parents and teachers of the children in kindergarten and elementary school must be knowledgeable in first aid practice and basic life support (BLS) training. Objectives: In the current study, our ambition is to evaluate the orientation level, knowledge, and attitudes around first aid and BLS training of kindergarten and elementary school teachers and parents in the city of Taif, Makkah region. Methods: A cross-sectional study in Taif, Saudi Arabia, targeted kindergarten and elementary school teachers and parents of students enrolled at these levels. There were 648 participants included in this study. The researchers assessed teachers’ and parents’ knowledge and attitudes around first aid and BLS using a validated, self-administered online questionnaire. Results: The study included 648 participants, including 248 (38.3%) teachers and 400 (61.7%) parents. The socio-demographic analysis showed that 412 (63.6%) are females and 233 (36.5%) are between the ages of 36 and 45 years. Approximately 142 (21.9%) participants reported previous training in the cardiopulmonary resuscitation (CPR) program, though more than half of them (53.5%) had outdated certificates (more than 2 years). The mean total knowledge for our study was 4.6 ± 1.4, with 22.4% of the participants being educated about first aid support and expressing a fair level of CPR foundations. Only a small percentage (2.3%) of the participants exposed a good and adequate theoretical level of knowledge around CPR skills and performance, while most of the contributors unveiled a poor level of knowledge (over 75%). There were no statistically significant differences between parents and teachers (*p* > 0.05). Finally, numerous participants (85%) appreciated training in the CPR program, and the most common motive was a “wish to avoid unnecessary death”. Conclusions: We concluded that a sizable portion of the contributors expressed a lack of proficiency in the fundamental CPR training knowledge and skills, pointing to an alarming public concern. Promisingly, a sizable percentage of participants expressed motivated attitudes toward CPR training. Therefore, additional study and data are required to effectively combat injury, with an emphasis on investigating causes and risk factors, burden and socioeconomic health determinants, community awareness level and desire to contribute, and accessibility for disseminating specific intervention strategies.

## 1. Introduction

In 1990, it was estimated that around 10% of the 51 million global deaths were attributed to injury occurrence. By 2020, this incidence was predicted to rise to 8.4 million yearly, making physical injury the leading cause of death among the general population, particularly children and youth. Moreover, the WHO’s 2008 World Report on Child Injury Prevention declared that nearly 900,000 children around the age of 17 and younger died from an injury in 2004, and most of these injuries were due to accidental and potentially avoidable harms. Therefore, injury has been defined as the “neglected disease of modern society”, an “invisible epidemic”, and “the principal public health problem in America” [1,2,3]. Physical injury is delineated as damage to the human body that exceeds its biological tolerance, resulting in a drought of crucial elements such as oxygen, or causing survival loss [1]. The sequel of the physical injury including death, lifelong disability, hospital admissions, or visiting the emergency department correlate largely with the intensity and the severity of the harm [4]. All these outcomes of physical injury result in significant healthcare costs and make injury an immeasurable health burden, mandating optimal intervention, better trauma care, and appropriate prevention measures [1]. Despite the reduction in the child unintentional injury (no intent to cause harm) death rate by 11% from 2010 through 2019, physical injury persists as the main cause of child and youth death in the United States. Indeed, it was reported that in 2019 the incidence of children and teenager death (age 0–19 year) in the US resulting from unintentional harms have reached over 7000 losses, equating to nearly 20 death each day [5]. Additionally, according to the CDC’s WISQARS™ database, unintentional injuries in the US are ranked as the third leading cause of infant death in 2021 and remained the leading cause of death in children and teens aged 1 to 19 years from 1981 to 2020 [5,6]. Likewise, a Canadian agency reported that injury is considered a major cause of death in all Canadians between the ages of 1 and 44. Furthermore, unintentional injury, especially, accounts for the leading cause of survival loss and hospital admissions in Canada, where children, youth, and rural people are the most susceptible populations to these injuries [1].

Two important factors that have contributed to the increase in the risk of injury are child development and behaviors. The curiosity for discovering, increasing mobility and activity levels, mouthing objects, attraction to dangerous items, inability to escape hazards, lack of experiences, and fearless attitudes are motivating factors for younger children, exposing them to harm [7]. On the other hand, limited experiences, emotions, cultures, parents, and peer influences contribute to the incidence of unintended injury in the youth group [8]. Based on the CDC 2021 report, transportation accidents; falls; burns and fires; suffocations; being struck (hitting) by humans, animals, or an inanimate object; poisoning; and drowning are the primary mechanisms of unintentional physical harms [9]. Suffocation is the most common mechanism of inadvertent injuries in the infant group, while motor vehicle accidents represent the most caused death-related unintended injuries in one year of age and older children [7,9]. These injuries were reported to happen commonly at home or on the school playground during free play activities [10,11]. Some injuries may be part of a bigger problem due to loss of consciousness. Cardiac arrest in pediatric groups is rare and most commonly due to respiratory arrest, as opposed to adult groups, when cardiac problems are more common [12]. The prognosis of in-hospital or out-of-hospital cardiopulmonary resuscitation (CPR) in pediatric groups is poor and may result in a devastating neurological deficit in the surviving patients [13,14]. According to the American Heart Association, if the heart stops beating, the first step in saving a life is to perform basic life support (BLS), starting with CPR. Both early initiation and high-quality delivered CPR increase the chances of survival in adult and pediatric groups [12,15]. To provide high-quality CPR, the provider should be well trained and understand the significance of time consumed performing CPR [14]. In order to rescue the patient’s life, alleviate the pain, terminate the progression of an injury, and obtain a favorable neurological outcome, correct CPR must be administered [14,16]. This emphasizes the importance of training school teachers and parents to perform CPR, as they are the main caregivers, to improve the outcome of out-of-hospital cardiac arrest [17].

Because injuries can result in devastating damage, disability, and death of children and youth, the implementation of a well-organized and effective child health prevention program would save over a thousand children’s lives each day [18]. Therefore, numerous studies have been conducted in different cities in Saudi Arabia to assess teachers’ knowledge and attitudes toward first aid. Al Enizi et al. evaluated the level of knowledge and awareness of CPR skills among secondary school teachers in the Al-Qassim region. The author found that the knowledge of CPR performance is inferior, with only 35.7% having CPR training [19]. A similar study in Al-Madinah Al-Munawwarah City revealed a non-satisfactory level of BLS knowledge among parents around common incidents and injuries, with only 34.6% having previous first aid training [20]. Moreover, subsequent reports on male teachers in Hail City and Riyadh Capital City showed that 58% to 74% of teachers, respectively, exhibited poor first aid knowledge and inappropriate reactions to frequent emergencies [21,22]. Similarly, a study in Al-Madinah Al-Munawwarah City reported that only 30.5% of female teachers were CPR certified [17]. A recent report in Buraidah City found that two-thirds of the participating mothers had an incorrect understanding of first aid training and its components. Nevertheless, a strong positive association between overall knowledge and attitude toward learning first aid was also found [23]. The following work in the Aseer Region assessed the knowledge of parents and the general population on pediatric first aid and trauma cases. This study found that participants had little knowledge of the first aid concept and procedures [24]. Two studies were conducted in Ethiopia in 2021 to evaluate teachers’ knowledge of first aid practice. One study was performed in kindergarten, and the other was performed in kindergarten and elementary school. Both studies have concluded that roughly 60% of teachers are unaware of the first aid procedure and approximately 75% and 66%, respectively, have favorable attitudes toward the first aid program [25,26]. Furthermore, a 2018 South African study on teachers’ knowledge demonstrated that 91% had not obtained CPR training [27]. Another report from Turkey shows that only one-third of the participants have completed the BLS training [28]. Communally, the comprehensive knowledge related to first aid and CPR training was found to be deficient among various settings and regions, yet the enthusiasm to learn these measurements was highly encouraging.

Unintentional and accidental injuries were reported in a considerable percentage of school-aged children, accounting for a critical cause of child morbidity and mortality [10,26]. Educators are the primary caregivers who can provide proper first aid skills to the affected children if they acquire a solid foundation in first aid training and CPR practice. Therefore, we aim in this work to evaluate the current level of knowledge around first aid practice and BLS of kindergarten and elementary school teachers and parents in Taif City, Saudi Arabia. The present study then aims to determine their attitudes and willingness to engage in first aid and CPR training programs. Our findings revealed that a small fraction of participants are aware of CPR skills, while the majority lack the essential understanding of this skill. Most participants were eager to take the CPR training and desired that it be free and mandatory for job requirements. Hence, a rapid and appropriate parental response would alleviate pain, increase the likelihood of the child’s survival, and considerably impact the outcome positively. Finally, we highly recommend that a caregiver (teacher or parent) receives sufficient first aid training.

## 2. Methods

### 2.1. Study Design and Setting

This is a descriptive cross-sectional study conducted in Taif in September 2022 among kindergarten and elementary school teachers and parents of students enrolled in these levels.

### 2.2. Study Population

The target population was parents and kindergarten and elementary teachers who live in Taif. Taif is a city located in the Makkah Region, the Western Area, of Saudi Arabia. Taif is located in Hijaz Mountains at 1879 m (6165 ft) high and has a total area of 321 km^2^ (124 sq mi). The population of Taif City is estimated in 2023 to be approximately 700,000 people, ranking it as the 6th most populous city in Saudi Arabia [29,30]. We included all parents with children under the age of 18 years and kindergarten and elementary teachers. We excluded parents with children above the age of 18 years and middle and high school teachers without children.

### 2.3. Sample Size

The sample size of this study was calculated using the following formula:

*n* = *z*^2^(1 − *p*)/*d*^2^

where *n* is the sample size, *z* is the statistic for a level of confidence (1.96% to 95% confidence level), *p* is the anticipated population proportion (50%) for the largest sample size, and *d* is precision (0.05 [5%]). The estimated sample size was 385; however, we increased it to 648.

### 2.4. Tools of Data Collection

We used a pre-validated and self-administered Arabic questionnaire from a previous study conducted by Alhejaili et al. [17] with permission from the author. The questionnaire was divided into six sections. The first section included seven questions about the participants’ socio-demographic information. The second section discussed the previous training states and is composed of three questions. The third section had 10 questions around BLS knowledge and skills. The fourth section included eight questions around attitudes toward learning and practicing CPR. In this section, positive attitudes toward learning CPR skills and performance were assessed among the participants by answering many questions about willingness and enthusiasm to take the training sessions, and by responding “Yes” were measured as a positive attitude. The fifth section asked nine questions around the barriers to performing CPR. The sixth section evaluated the resuscitation experience in BLS and is composed of two questions. A group of data collectors distributed the questionnaire via social media platforms. The format of the questionnaire is included at the end of the manuscript as an Appendix A. The total knowledge level related to CPR was calculated by adding the scores obtained for each knowledge item, where each correct response received a score of 1 and the wrong response received no score. Thus, one participant could receive a maximum score of 10 and a minimum of 0. Next, the total scores were divided into three levels based on the percentage of scores obtained to assess the knowledge level. Participants with a score of >75% were considered to have a “good” knowledge level, those with a score of 50–74.9% were considered “fair,” and those with a score of less than 50% were considered “poor”.

### 2.5. Statistical Analysis

The data collected were cleaned (the data from the questionnaire were entered into a database (Microsoft Excel for Mac, version 16.32)) and then analyzed using SPSS version 23 (IBM Corp. Chicago, IL, USA). The data analysis was carried out by an independent biostatistician. Categorical variables were presented as frequencies and percentages. Continuous variables were expressed using mean and standard deviations. Pearson’s Chi-square test was used to evaluate the statistical relationship between categorical variables. A *p*-value of ≤0.05 was considered statistically significant.

## 3. Results

Unintended injuries are the leading cause of death in children and teens. As most of these accidental injuries happen at home and school, parents and teachers must be well trained in BLS foundations and act quickly and appropriately to increase the likelihood that a child will survive. Therefore, we aimed to assess the orientation level, knowledge, and attitudes of kindergarten and elementary school teachers and parents toward first aid measurements in the city of Taif. In the current work, we recruited 648 participants who met the study criteria, with 248 (38.3%) teachers and 400 (61.7%) parents. As illustrated in Table 1, the socio-demographic analysis described the following characteristics of our participants, including the fact that 412 (63.6%) were females, 233 (36%) were between the ages of 36 and 45 years, 629 (96.9%) were Saudi citizens, 483 (74.5%) were married, 473 (73%) had a graduate level of education, 387 (59.7%) worked in the educational field, and 39 (6%) worked in the healthcare field. Importantly, our analysis found that a low percentage of the participants, around 142 (21.9%), received former training in CPR, with 44 (31%) obtaining the training from a private institution, 35 (24.6%) receiving the training from the Red Crescent, and 38 (26.8%) having the courses from organized classes that were arranged in their schools. Remarkably, our result further showed that more than half (53.5%) of contributors who acquired previous CPR courses (*n* = 142) revealed outdated training, which is over 2 years, whereas 14.8% of contributors (from the same group, *n* = 142) had recent training within 0–6 months. Together, most of the participants in our study showed a lack of CPR training, and those contributors who attended CPR courses previously were found to have outdated certification in most of the cases.

**Figure 1:**
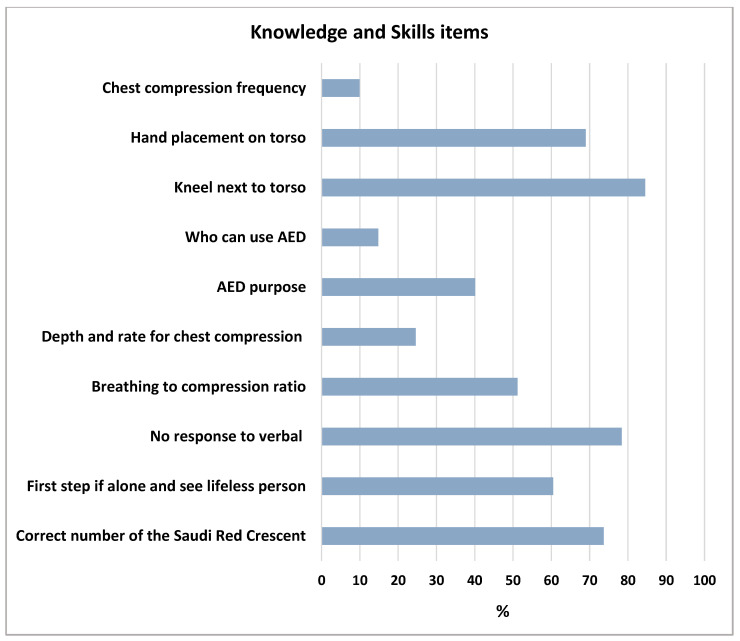
Graphic bars show the percentages of the correct responses to the knowledge and skills elements of the CPR assessment.

Next, to evaluate the current understanding and information around CPR skills, a pre-validated questionnaire containing many sections with different items (as previously described in the Method section, Tools of Data Collection) was distributed among the participants. The responses of participants for various items related to CPR knowledge and skills are shown in Table 2. Our analysis found that approximately 73.3% of the participants know the correct contact number of the Saudi Red Crescent. Furthermore, around 60.5% of the participants realize what to do when alone and coming across a lifeless adult person. When asked what they do when a patient is breathing but not responding to verbal stimuli, most of the participants (78.4%) identified the correct action (i.e., place the patient in a recovery position and call for an ambulance). Notably, 51.2% of participants comprehend the combinations of chest compressions and ventilation techniques to perform CPR. In addition, a small percentage (24.6%) identified the appropriate depth and rate ratio for chest compression when performing CPR. Our findings also showed that around 40.1% of participants understand the purpose of an automated external defibrillator (AED), and around 14.8% of participants can distinguish who is allowed to use an AED. The skill assessment showed that nearly 84.5% of participants know that respondents should kneel next to the torso when performing CPR, as well as around 69% recognize the correct hand placement on the torso. We also found that a low percentage (9.9%) of the participants defined the correct chest compression frequency (two compressions/1 s) when performing CPR, as presented in Table 2 and Figure 1. Collectively, these findings demonstrated a deficiency in the comprehensive understanding, knowledge, and awareness of CPR elements and performance among the participants.

The knowledge and experience of different skill elements when performing CPR were assessed among the participants. These items include chest compression frequency, hand placement on the torso, kneeling next to the torso, who can use an AED, the purpose of an AED, depth and rate for chest compression, breathing to compression ratio, no response to verbal stimuli, first step if you are alone and see a lifeless person, and the correct number of the Saudi Red Crescent. CPR, cardiopulmonary resuscitation; AED, automated external defibrillator.

**Figure 2:**
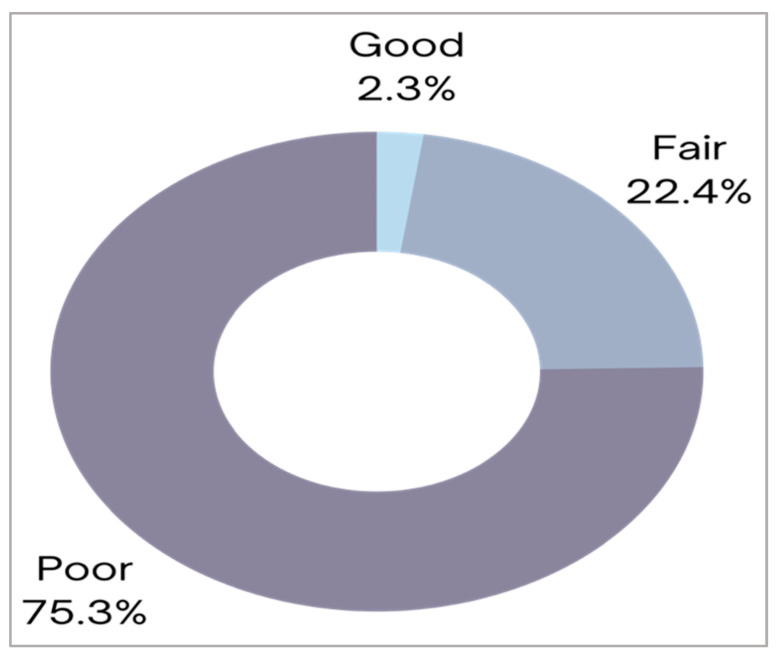
Schematic diagram that presents the knowledge levels of different CPR training skills among the participants based on different sociodemographic characteristics.

To scrutinize comprehensively the awareness and information level related to CPR knowledge and skills among the participants, we next categorized the knowledge of contributor groups into different levels defined as good, fair, and poor knowledge participants. To attain this purpose, the questions related to CPR knowledge and skills (formerly explained in Table 2) were scored and calculated, based on sociodemographic features, by adding the scores obtained for each knowledge item, where each correct response received a score of 1 and the wrong response received no score (refer to Method section, Tools of Data Collection). Participants with a score of >75% were considered to have a “good” knowledge level, those with a score of 50–74.9% were considered “fair”, and those with a score of less than 50% were considered “poor” knowledge participants (Figure 2).

Different questions related to the basic knowledge of CPR techniques and skills were asked of the participants. The total knowledge level related to CPR was calculated by adding the total scores, as each item/question has a specific score. The scores are divided into good responses if the participant scores >75%, fair responses when the score is 50–74.9%, and is considered as poor knowledge if the score is <50%. The analysis showed that only 15 (2.3%) participants had a “good” knowledge level, whereas most of the participants 488 (75.3%) revealed a “poor” knowledge level.

Furthermore, the highest level of knowledge, i.e., good level category, was obtained by the participants who received CPR training as compared to the other groups, as shown in Table 3. Indeed, these participants with a good knowledge level (total *n* = 15) were found to have mostly (*n* = 11/15) previous CPR training. Conversely, the contributors with a poor knowledge level (total *n* = 488) showed predominately (*n* = 411/488) absence of CPR training, thus resulting in a statistically significant difference (*p* < 0.001) between the examined groups. Among the other sociodemographic parameters, our analysis disclosed no statistically significant differences observed between knowledge levels and time since previous CPR training (*p* > 0.05). Moreover, when we assessed the differences in knowledge level between parents and teachers, we also found no statistically significant differences (*p* > 0.05). Similarly, there were no statistically significant differences in knowledge levels between genders, age groups, nationalities, educational levels, and job fields (Table 3). Collectively, as graphed in Figure 2 and Table 3, our analysis showed that only a small percentage, around 2.3% (i.e., 15 participants), unveiled a “good” knowledge level, whereas the majority (75.3%, i.e., 488 participants) had a poor knowledge level of CPR performance and skills. These findings are affected significantly by whether a participant had acquired former CPR training or not.

**Figure 3:**
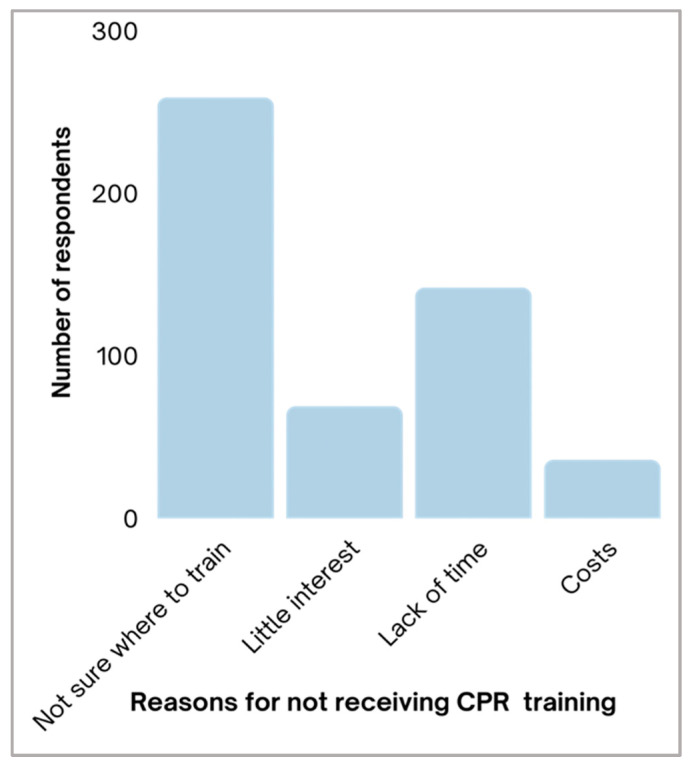
Graphic bars reveal the different reasons for not having received CPR training previously among 506 CPR-untrained respondents.

As it was demonstrated by our analysis that most of the participants did not have CPR training, next, we were ambitious to delineate the reasons for not taking these courses. A lack of knowledge of the CPR training centers accounted for the most common cause of not obtaining these classes (51.2%). This was followed by a lack of time (28.1%), diminutive interest (13.6%), and finally the cost of the enrollment fee (7.1%) (Figure 3). When we asked the participants what their perception is regarding the reason that makes an individual hesitate to start CPR, nearly 60.6% reported that it was due to a “lack of proper knowledge and skills”. Most of the participants (85%) demanded more training in CPR, and the most common reason for that was a “wish to avoid unnecessary death”. Our study also revealed that most participants (87.7%) would attend CPR training if it was costless. Notably, 41.5% of contributors believed that training should be made mandatory for every job. Indeed, 50.6% of the participants believed that CPR should be required for obtaining a teaching job, specifically, and most participants (80.6%) believed that AEDs should be provided in every school. Finally, around 42.6% reported seeing actual CPR, whereas only 9.1% of contributors performed CPR (Table 4). Jointly, this analysis demonstrated the positive attitudes expressed by the contributors for having CPR training as well as mandating this item for job requirements.

Lack of knowledge of the center to obtain CPR training presented the most common reason for not taking CPR training among the participants (259, 51.2%), followed by a lack of time (142, 28.1%), and lack of interest (69, 13.6%), and the least common reason was related to the course’s fee, which accounts for 36 participants or 7.1%.

## 4. Discussion

Unintentional injuries among children and youth continue to be the leading cause of death among these populations and, further, place an extremely high cost on the public health system. The primary places of these injuries are at home and at school, thus necessitating that caregivers must obtain the foundational knowledge of life support courses. In this study, we aimed to measure kindergarten and elementary school teachers’ and parents’ knowledge and attitudes toward first aid and BLS services in Taif City, Saudi Arabia. Our work found that a small fraction (24.7%) of the participants were educated about first aid support and only 2.3% of them had a good and adequate theoretical level of knowledge. In alignment with our results, two previous studies conducted among teachers in Al-Madinah Al-Munawwarah City and Al-Qassim Region, Saudi Arabia, have reported similar findings [17,19]. We also found that 40.1% of participants recognized the purpose of an AED and 14.8% knew who is allowed to use an AED. These figures are better than those obtained from a previous study in Al-Madinah Munawwarah City that showed that 16.6% of contributors understood the aim of an AED and only 8.3% of them knew who can use it [17]. Additionally, we did not find a significant difference (*p* = 0.682) in knowledge levels between teachers and parents, which might be attributed to a lack of awareness. Furthermore, our results showed that female respondents had less knowledge than male respondents. This may explain why males in Saudi Arabia are more likely to be involved in rescuing individuals during accidents and sudden cardiac arrests [31]. Likewise, there was no significant relationship between age and knowledge (*p* = 0.244), in contrast to a study conducted in Athens, Greece, which reported that the age of the teachers correlated positively with the ratio of incorrect answers [32].

Our analysis of parents’ knowledge levels revealed that parents employed in the healthcare sector have a higher level of knowledge (fair knowledge, 33.3%) than parents engaging in other fields (education, 20.9%; no job, 24.8%; and others, 19.2%). This finding explained the potential role of healthcare services in educating their employees regarding fundamental life support skills. In the present study, over 21.9% of respondents had received CPR training in the past; comparable results were seen in former studies conducted in Buraidah and Riyadh cities, Saudi Arabia, wherein they identified that 23.9% of mothers and 26.4% of male schoolteachers had received CPR courses [22,23]. On the other hand, in Isparta, Turkey, 73.6% of the kindergarten instructors had acquired a first aid course. We predict that the fact that first aid is covered in driving courses accounts for the large number of teachers who have received this training [33]. Moreover, we found that first aid training and knowledge level are significantly correlated; knowledge level is enhanced by CPR classes. Similarly to this, earlier research found a substantial correlation between prior CPR courses and knowledge level [21,25,27]. Nevertheless, a study carried out in Al-Qassim discovered no connection between training and first aid expertise [19]. This may be because the CPR training was completed more than two years prior to the study’s execution. These findings underlined how crucial it is to regularly update knowledge by participating in CPR training. Notably, our study’s findings showed that 78.1% of participants had never previously taken CPR training. The most prevalent excuse (51.2%) was not knowing the location of the training centers. The same basic rationale was also found in earlier research that was carried out in Al-Madinah Munawwarah City [17]. This demonstrates the demand for more community CPR training accessibility and marketing and implies that disclosing details about training sites might boost participation rates. Currently, virtual CPR training is a promising new approach to promoting accessibility to CPR training. Several studies have shown that virtual CPR training can be effective for improving knowledge and skills and for increasing the willingness of bystanders to perform CPR [34]. A study by Nas et al. showed long-term knowledge retention and willingness to perform CPR following virtual CPR training [35].

According to the findings of this study, the overall attitude toward CPR training is promising. A similar outcome was obtained from studies among teachers in Gondar, Ethiopia, and Riyadh, Saudi Arabia [22]. Furthermore, Bassam et al. [23] revealed that most participating parents and teachers had a favorable attitude toward first aid training. In contrast, according to a study on maternal knowledge and attitude toward first aid in Buraidah, more than half of the evaluated mothers showed undesirable attitudes toward first aid practice [23]. Multiple studies explored the barriers to performing CPR by bystanders. A study was conducted in Columbus, Ohio, to examine the prevalence of bystander CPR in neighborhoods with high rates of cardiac arrest and low rates of CPR training [36]. Through the study, researchers identified several key barriers to learning CPR: financial, informational and knowledge gaps, motivational, fear of legal consequences, emotional issues, and situational concerns [36]. A separate systematic review delved into the barriers and facilitators to bystander CPR in disadvantaged communities [37]. The findings of this review identified three main components that acted as barriers: willingness to learn or perform CPR, confidence to perform CPR, and self-reported likelihood of performing CPR [37]. The fear of contracting infectious diseases through mouth-to-mouth breathing also created challenges for bystander CPR performance [38]. The outbreak of COVID-19 has also had an impact on the public’s level of training and confidence when it comes to performing CPR. With people worried about the potential for infection and following additional safety measures, it may have discouraged some individuals from providing CPR during the pandemic [39]. Chest compression-only CPR (CC-CPR) can be an alternative for people who are hesitant to perform mouth-to-mouth breathing. Multiple studies showed that CC-CPR can deliver good quality CPR [40,41], but this can be difficult to implement in pediatric CPR since most of the arrests are due to respiratory causes.

Furthermore, a significant percentage of participants (85%) were willing to learn CPR to prevent unnecessary, deaths and most participants (87.7%) would attend CPR training if it were provided at no cost. Al-Madinah’s study findings are consistent with our findings [17]. This suggests that providing free CPR training could effectively increase the number of people trained in CPR in Al-Madinah and other regions. Community awareness of the importance of first aid and CPR is shown by the significant proportion (>80%) of respondents who thought CPR training should be mandatory for all citizens. Although previous research has shown that more than 90% of people think CPR training should be mandatory for teachers, only 50% of our respondents agree that it should be a prerequisite for teacher certification [28]. In Saudi Arabia, neither the teacher certification program nor the school curriculum includes CPR instructions. Giving teachers CPR training, however, can improve their capacity and skills to save lives in both school-related and non-school-related crises, such as those that arise at home or in public settings. The incorporation of a comprehensive and integrated surveillance system combining efforts of education, enforcement/legislation, and engineering/environment would potentially reduce the injury burden by 40% [1]. For example, Sweden is among the countries that have achieved the lowest injury rates worldwide by implementing solid policies and regulations to prevent the occurrences of injuries [18]. Therefore, an inclusive understanding of all risk factors of unintentional harm and fortifying the engagement of operative strategies to prevent them are necessitated.

There are a few restrictions on this study. One is the disparity in participation rates between men and women, which may be skewed due to the survey’s distribution among female groups and may impair the accuracy of knowledge estimation. The fact that the teachers’ percentage is lower than the parents’ is another drawback. Additionally, the school was chosen at random, thus it may be challenging to extrapolate the findings to all Taif City schools. Finally, the fact that the survey is online causes participants to become hesitant, which causes them to look for the right answers to some survey questions online.

## 5. Conclusions and Recommendations

Collectively, unintentional injuries among children and youth continue to generate unacceptable high public health burdens, albeit substantial amendments can be effectively delivered. To reduce discomfort, enhance the possibility that the injured kid will survive, and significantly improve the prognosis, prompt and effective caregiver intervention is essential. The results of the current study showed that while the majority of participants lacked an in-depth understanding of CPR training techniques, the minority demonstrated a good and appropriate theoretical level of knowledge about CPR abilities and performance, suggesting a worrying public issue. Promisingly, a significant percentage of participants showed encouraging responses toward acquiring CPR training. Therefore, to successfully battle injury, more research and evidence are needed, focusing on exploring causes and risk factors, burden and social health determinants, community awareness level and willingness to contribute, and accessibility for delivering specific intervention measures. The following are some of our suggestions for raising awareness and enhancing the provision of basic life support capacities:

- Implementing evidence-based first aid training and practice that address the community determinants and inserting it as a vital part of primary healthcare services. This would give citizens the knowledge and skills needed to help in an emergency.
- Organizing an injury prevention program that consists of good strategies, policies, coordination, surveillance, education, and social marketing.
- Providing proper education to the public, parents, and teachers about different injury risk factors and the influencing behaviors through social media and school-based curriculums.
- Advocating for the integration of first aid and CPR programs into the school curriculum with organization of more first aid training workshops for teachers.
- Encouraging the inclusion of first aid training certificates into job requirements, especially for educators.
- Providing a safe and controlled environment for children and youth to learn, play, and live.

## Figures and Tables

**Table 1:** Different sociodemographic details of the participants.

Sociodemographic Characteristics		*n*	%
Gender	Female	412	63.6
	Male	236	36.4
Age	<25	74	11.4
	25–35	122	18.8
	36–45	233	36.0
	46–55	172	26.5
	56–60	30	4.6
	>60	17	2.6
Nationality	Saudi	629	96.9
	Non-Saudi	19	3.1
Marital status	Single	125	19.3
	Married	483	74.5
	Divorced	24	3.7
	Widowed	16	2.5
Participants group	Teachers	248	38.3
	Parents	400	61.7
Educational qualification	Primary	21	3.2
	Secondary	82	12.7
	Middle	15	2.3
	Graduate	473	73.0
	Post-graduate	57	8.8
Job sector	Educational field	387	59.7
	Health field	39	6.0
	Not employed	149	23.0
	Other	73	11.3
Had previous training in CPR	No	506	78.1
	Yes	142	21.9
Training center (*n* = 142)	In school	38	26.8
	Private institution	44	31.0
	Red Crescent	35	24.6
	Another workplace	25	17.6
Time since CPR training done (*n* = 142)	0–6 months	21	14.8
	7–12 months	23	16.2
	13–24 months	22	15.5
	>2 years	76	53.5

**Table 2:** Questions related to CPR knowledge and skills.

Questions		*n*	%
The correct number of the Saudi Red Crescent:	997 *	475	73.3
	998	112	17.3
	999	61	9.4
You are alone and come across an apparently lifeless adult person. What do you do?	Check for consciousness, secure airways, and check if the patient is breathing *	392	60.5
	Check for pulse	206	31.8
	Immediately start chest compressions	50	7.7
It turns out the patient is breathing but shows no response to verbal stimuli. What do you do?	Check for pulse	68	10.5
	Immediately start chest compressions	72	11.1
	Put the patient in recovery position and call for ambulance *	508	78.4
You decide to perform CPR. Which of the following combinations of chest compressions and ventilations would you choose?	2 rescue breathings and 30 chest compressions	172	26.5
	30 chest compressions and 2 rescue breathings *	332	51.2
	30 chest compressions and 5 rescue breathings	144	22.2
What is the appropriate depth and rate for chest compressions?	4–5 cm and 100/min	71	50.0
	At least 5 cm and 100–120/min *	35	24.6
	I don’t know	36	25.4
What is the purpose of the automated external defibrillator?	To analyze the heart rhythm	24	16.9
	To analyze the heart rhythm and if necessary to give an electric shock *	57	40.1
	To give cardiac massage	25	17.6
	I don’t know	36	25.4
Who is allowed to use an (automated external defibrillator) AED?	Every citizen *	21	14.8
	Only emergency personnel	52	36.6
	Only skilled people	69	48.6
Does the respondent kneel next to the torso?	No	22	15.5
	Yes *	120	84.5
How is the hand placement on the torso?	Used one hand as a fist	8	5.6
	Two hands next to each other	9	6.3
	Place one hand above the other with palms of the hands downwards *	98	69.0
	Cross the fingers of both hands	20	14.1
	Not able to place hands	1	.7
	Used of one hand	6	4.2
The appropriate chest compression frequency to be done in CPR?	<1 compression/1 s	17	12.0
	1 compression/1 s	70	49.3
	1 compression/2 s	24	16.9
	2 compressions/1 s *	14	9.9
	>2 compressions/1 s	10	7.0
	Irregular compression	7	4.9

* Indicates the correct answer for each question.

**Table 3:** Knowledge level based on different sociodemographic characteristics.

Sociodemographic Characteristics			Knowledge Level			*p* Value *
			Good	Fair	Poor	
Groups	Teacher	*n*	6	51	191	0.682
		%	2.4%	20.6%	77.0%	
	Parents	*n*	9	94	297	
		%	2.3%	23.5%	74.3%	
Gender	Female	*n*	9	81	322	0.081
		%	2.2%	19.7%	78.2%	
	Male	*n*	6	64	166	
		%	2.5%	27.1%	70.3%	
Age	<25	*n*	2	20	52	0.244
		%	2.7%	27.0%	70.3%	
	25–35	*n*	6	24	92	
		%	4.9%	19.7%	75.4%	
	36–45	*n*	6	55	172	
		%	2.6%	23.6%	73.8%	
	46–55	*n*	0	39	133	
		%	0.0%	22.7%	77.3%	
	56–60	*n*	0	5	25	
		%	0.0%	16.7%	83.3%	
	>60	*n*	1	2	14	
		%	5.9%	11.8%	82.4%	
Nationality	Saudi	*n*	15	140	474	0.742
		%	2.4%	22.3%	75.4%	
	Non-Saudi	*n*	0	5	14	
		%	0.0%	26.3%	73.7%	
Educational level	Primary	*n*	0	8	13	0.430
		%	0.0%	38.1%	61.9%	
	Middle	*n*	1	6	8	
		%	6.7%	40.0%	53.3%	
	Secondary	*n*	2	17	63	
		%	2.4%	20.7%	76.8%	
	Graduate	*n*	11	102	360	
		%	2.3%	21.6%	76.1%	
	Post-graduate	*n*	1	12	44	
		%	1.8%	21.1%	77.2%	
Job field	Educational field	*n*	8	81	298	0.282
		%	2.1%	20.9%	77.0%	
	Health field	*n*	2	13	24	
		%	5.1%	33.3%	61.5%	
	Not employed	*n*	2	37	110	
		%	1.3%	24.8%	73.8%	
	Other	*n*	3	14	56	
		%	4.1%	19.2%	76.7%	
Previous training in CPR	No	*n*	4	91	411	<0.001 *
		%	0.8%	18.0%	81.2%	
	Yes	*n*	11	54	77	
		%	7.7%	38.0%	54.2%	
Time since received CPR training (*n* = 142)	0–6 months	*n*	3	6	12	0.453
		%	14.3%	28.6%	57.1%	
	7–12 months	*n*	3	11	9	
		%	13.0%	47.8%	39.1%	
	13–24 months	*n*	2	8	12	
		%	9.1%	36.4%	54.5%	
	>2 years	*n*	3	29	44	
		%	3.9%	38.2%	57.9%	

* Indicates the value is significant.

**Table 4:** Perceptions and attitudes towards CPR.

Perceptions and Attitudes		*n*	%
Reason for not receiving CPR training (*n* = 506)	Costs	36	7.1
	Lack of time	142	28.1
	Little interest	69	13.6
	Not sure where to attend course	259	51.2
Perception regarding reasons that make a person hesitate to start CPR	Afraid of contagious diseases through mouth-to-mouth breath	40	6.2
	Afraid of legal consequences	77	11.9
	Causing potential harm to the person in need	97	15.0
	Emotional reasons	41	6.3
	Lack of proper knowledge and skills	393	60.6
Require more training in CPR	No	94	14.5
	Yes	554	85.5
Motive for doing more training (*n* = 554)	Heart disease within the family	46	8.3
	Wish of avoiding unnecessary death	282	50.9
	Other reasons	226	40.8
Would like to attend CPR if it is of no cost	No	80	12.3
	Yes	568	87.7
Think CPR training should be mandatory	Yes, at school	235	36.3
	Yes, to obtain a driving license	39	6.0
	Yes, training should be mandatory in every job	269	41.5
	No, CPR training should be optional	105	16.2
CPR training part of the educational plan in the school (answered only by the teachers) (*n* = 248)	No	145	58.5
	Yes	103	41.5
Think that CPR training should be a requirement for obtaining a teaching job	No	320	49.4
	Yes	328	50.6
Think that an automated external defibrillator should be available in every school	No	126	19.4
	Yes	522	80.6
Seen doing real CPR	No	372	57.4
	Yes	276	42.6
Performed CPR in real	No	589	90.9
	Yes	59	9.1

## Data Availability

The datasets used and/or analyzed during the current study are available from the corresponding author upon reasonable request.

## Appendix A

A. Demographic data

1. Gender
  - Male
  - Female
2. Age
3. Nationality
  - Saudi
  - Non-Saudi
4. Where do you live?
  - In Taif
  - Outside Taif
5. Marital status
  - Single
  - Married
  - Divorced
  - Widowed
6. Educational Qualification
  - Primary
  - Secondary
  - Middle
  - Graduate
  - Post-graduate
7. Job Sector
  - Educational field
  - Health field
  - Not employed
  - Other

B. Training status

1. Previous CPR training?
  - Yes
  - No
2. Where and how have the respondents received their CPR training?
  - In the school
  - Red Cross
  - Private organization
  - Other work
  - Others
3. Time since previous CPR training?
  - Between 0 and 6 months
  - Between 7 and 12 months
  - Between 13 and 24 months
  - More than 2 years

C. Knowledge assessment

1. What is the correct emergency phone number?
  - 999
  - 998
  - 997
2. You are alone and come across a lifeless adult person. What do you do?
  - Immediately start chest compressions
  - Check for consciousness, secure airways, and check if the patient is breathing
  - Check for pulse
3. It turns out the patient is breathing but shows no response to verbal stimuli. What do you do?
  - Immediately start chest compressions
  - Put the patient in a recovery position and call for an ambulance
  - Check for pulse
4. You decide to perform CPR. Which of the following combinations of chest compressions and ventilations would you choose?
  - 30 chest compressions: 2 rescue breathings
  - 30 chest compressions: 5 rescue breathings
  - 2 rescue breathings: 30 chest compressions
5. How deep and how fast would you perform chest compressions?
  - 4–5 cm and 100/min
  - At least 5 cm and 100–120/min
  - No answer
6. What is the purpose of an automated external defibrillator (AED)?
  - To analyze the heart rhythm
  - To analyze the heart rhythm and give an electric shock if necessary
  - To give cardiac massage
  - I do not know
7. Who is allowed to use an AED?
  - Every citizen
  - Only skilled people
  - Only emergency personnel

D. Skills assessment

1. Does the respondent kneel next to the torso?
  - Yes
  - No
2. How is the hand placement on the torso?
  - Use one hand
  - Place one hand above the other with the palms downward
  - Use one hand as a fist
  - Cross the fingers of both hands
  - Two hands next to each other
  - Not able to place hands
3. Chest compression frequency
  - Less than one press/s
  - One press/2 s
  - One press/s
  - Two presses/s
  - More than two presses/s
  - Irregular press

E. Attitude toward training (the reasons to attend the BLS training and to learn BLS).

1. If you had no previous CPR training, what is the reason?
  - Little interest
  - Little time
  - Not sure where to attend the course
  - Costs
  - No answer
2. What reason makes people afraid to apply BLS to victims?
  - Afraid of contagious diseases through the mouth-to-mouth breath
  - Causing potential harm to the person in need
  - Afraid of legal consequences
  - Emotional factors
  - Lack of proper knowledge and skills
3. Do you want more training?
  - Yes
  - No
4. If you want more CPR training, what is the reason?
  - Heart disease within the family
  - Wish to avoid unnecessary death
  - Other reason or no answer
5. Would you be willing to take a free CPR course if it were offered?
  - Yes
  - No
6. Do you think CPR training should be mandatory?
  - Yes, at school.
  - Yes, to obtain a driver’s license.
  - Yes, training should be mandatory in every job.
  - No, CPR training should be optional.
7. Is CPR already part of the educational curriculum?
  - Yes
  - No
8. Do you think CPR training should be required for teacher certification?
  - Yes
  - No
9. Do you think each school should have an AED?
  - Yes
  - No

F. Resuscitation experiences

1. Have you observed CPR on a collapsed patient?
  - Yes
  - No
2. Have you participated in CPR before?
  - Yes
  - No

## References

[1] Yanchar N.L., Warda L.J., Fuselli P. (2012). Child and youth injury prevention: A public health approach. Paediatr. Child. Health.

[2] Murray C.J., Lopez A.D. (1997). Mortality by cause for eight regions of the world: Global Burden of Disease Study. Lancet.

[3] Murray C.J., Lopez A.D. (1997). Alternative projections of mortality and disability by cause 1990-2020: Global Burden of Disease Study. Lancet.

[4] Razzak J.A., Sasser S.M., Kellermann A.L. (2005). Injury prevention and other international public health initiatives. Emerg. Med. Clin. N. Am..

[5] West B.A., Rudd R.A., Sauber-Schatz E.K., Ballesteros M.F. (2021). Unintentional injury deaths in children and youth, 2010–2019. J. Saf. Res.

[6] Ballesteros M.F., Webb K., McClure R.J. (2017). A review of CDC’s Web-based Injury Statistics Query and Reporting System (WISQARS™): Planning for the future of injury surveillance. J. Saf. Res..

[7] Dellinger A., Gilchrist J. (2019). Leading Causes of Fatal and Nonfatal Unintentional Injury for Children and Teens and the Role of Lifestyle Clinicians. Am. J. Lifestyle Med..

[8] Schwebel D.C., Gaines J. (2007). Pediatric unintentional injury: Behavioral risk factors and implications for prevention. J. Dev. Behav. Pediatr..

[9] Albedewi H., Al-Saud N., Kashkary A., Al-Qunaibet A., AlBalawi S.M., Alghnam S. (2021). Epidemiology of childhood injuries in Saudi Arabia: A scoping review. BMC Pediatr..

[10] Scheidt P.C., Harel Y., Trumble A.C., Jones D.H., Overpeck M.D., Bijur P.E. (1995). The epidemiology of nonfatal injuries among US children and youth. Am. J. Public Health.

[11] Alkon A., Genevro J.L., Tschann J.M., Kaiser P., Ragland D.R., Boyce W.T. (1999). The epidemiology of injuries in 4 child care centers. Arch. Pediatr. Adolesc. Med..

[12] Duff J.P., Topjian A.A., Berg M.D., Chan M., Haskell S.E., Joyner B.L., Lasa J.J., Ley S.J., Raymond T.T., Sutton R.M. (2019). 2019 American Heart Association Focused Update on Pediatric Advanced Life Support: An Update to the American Heart Association Guidelines for Cardiopulmonary Resuscitation and Emergency Cardiovascular Care. Circulation.

[13] Mick N.W., Williams R.J. (2020). Pediatric Cardiac Arrest Resuscitation. Emerg. Med. Clin. N. Am..

[14] Kurt F., Kendirli T., Gündüz R.C., Kesici S., Akça H., Şahin Ş., Kalkan G., Derbent M., Tuygun N., Ödek Ç. (2018). Outcome of out-of-hospital cardiopulmonary arrest in children: A multicenter cohort study. Turk. J. Pediatr..

[15] Highlights of the 2020 AHA Guidelines Update for CPR and ECC. American Heart Association. https://cpr.heart.org/en/resuscitation-science/cpr-and-ecc-guidelines.

[16] Zideman D.A., De Buck E.D., Singletary E.M., Cassan P., Chalkias A.F., Evans T.R., Hafner C.M., Handley A.J., Meyran D., Schunder-Tatzber S. (2015). European Resuscitation Council Guidelines for Resuscitation 2015 Section 9. First aid. Resuscitation.

[17] Alhejaili A.S., Alghamdi R.A., Al-Dubai S.A.R. (2020). Knowledge and attitude of basic life support skills among female school teacher in Al-Madinah, Saudi Arabia. J. Fam. Med. Prim. Care.

[18] Peden M., Oyegbite K., Ozanne-Smith J., Hyder A.A., Branche C., Rahman A., Rivara F., Bartolomeos K. (2008). WHO Guidelines Approved by the Guidelines Review Committee. World Report on Child Injury Prevention.

[19] Al Enizi B.A., Saquib N., Zaghloul M.S., Alaboud M.S., Shahid M.S., Saquib J. (2016). Knowledge and Attitudes about Basic Life Support among Secondary School Teachers in Al-Qassim, Saudi Arabia. Int. J. Health Sci..

[20] Al-Johani A.A.S., Sabor S., Aldubai S.A.R. (2018). Knowledge and practice of first aid among parents attending Primary Health Care Centers in Madinah City, Saudi Arabia, A Cross Sectional Study. J. Fam. Med. Prim. Care.

[21] Alshammari K.O. (2021). Assessment of knowledge, attitude, and practice about first aid among male school teachers in Hail city. J. Fam. Med. Prim. Care.

[22] AlYahya I.A., Almohsen H.A., AlSaleem I.A., Al-Hamid M.M., Arafah A.M., Al Turki Y.A., Aljasser A.A., Alkharfi M.A. (2019). Assessment of knowledge, attitude, and practice about first aid among male school teachers and administrators in Riyadh, Saudi Arabia. J. Fam. Med. Prim. Care.

[23] Bassam S.E.A. (2022). Evaluate Maternal Knowledge and Attitude Regarding First Aid Among their Children in Buraidah City, Saudi Arabia Kingdom (KSA). Med. Arch..

[24] Wani J.I., Almushayt N.O., Abbag W.F., Buhran L.A., Nadeem M. (2022). Pediatric first aid, trauma knowledge, and attitude among parents and general population in Aseer region, Southern Saudi Arabia. SAGE Open Med..

[25] Ganfure G., Ameya G., Tamirat A., Lencha B., Bikila D. (2018). First aid knowledge, attitude, practice, and associated factors among kindergarten teachers of Lideta sub-city Addis Ababa, Ethiopia. PLoS ONE.

[26] Workneh B.S., Mekonen E.G., Ali M.S. (2021). Determinants of knowledge, attitude, and practice towards first aid among kindergarten and elementary school teachers in Gondar city, Northwest Ethiopia. BMC Emerg. Med..

[27] Ojifinni K., Motara F., Laher A.E. (2019). Knowledge, Attitudes and Perceptions Regarding Basic Life Support Among Teachers in Training. Cureus.

[28] Dursun A., Özsoylu S., Emeklioğlu B., Akyıldız B.N. (2018). Evaluating the basic life support knowledge among schoolteachers: A cross-sectional survey in Kayseri, Turkey. Turk. J. Pediatr..

[29] (2016). Britannica TEoElocatiSAEB. https://www.britannica.com/topic/list-of-cities-and-towns-in-Saudi-Arabia-2066809.

[30] Wikipedia Contributors T. Wikipedia, The Free Encyclopedia. https://en.wikipedia.org/w/index.php?title=Taif&oldid=1158268174.

[31] Alanazy A.R.M., Fraser J., Wark S. (2022). Emergency medical services in rural and urban Saudi Arabia: A qualitative study of Red Crescent emergency personnel’ perceptions of workforce and patient factors impacting effective delivery. Health Soc. Care Community.

[32] Patsaki A., Pantazopoulos I., Dontas I., Passali C., Papadimitriou L., Xanthos T. (2012). Evaluation of Greek high school teachers’ knowledge in basic life support, automated external defibrillation, and foreign body airway obstruction: Implications for nursing interventions. J. Emerg. Nurs..

[33] Sönmez Y., Uskun E., Pehlivan A. (2014). Knowledge levels of pre-school teachers related with basic first-aid practices, Isparta sample. Turk. Pediatri Ars..

[34] Cross M., Harlow E., Morrison S.R., Place M., Sutherland M., Thomas J., Leslie S.J. (2019). Bystander CPR training: Is non-classroom based CPR training as effective as a classroom based approach? A systematic review of randomised controlled trials. Rural Remote Health.

[35] Nas J., Thannhauser J., Konijnenberg L.S.F., van Geuns R.M., van Royen N., Bonnes J.L., Brouwer M.A. (2022). Long-term Effect of Face-to-Face vs Virtual Reality Cardiopulmonary Resuscitation (CPR) Training on Willingness to Perform CPR, Retention of Knowledge, and Dissemination of CPR Awareness: A Secondary Analysis of a Randomized Clinical Trial. JAMA Netw. Open.

[36] Sasson C., Haukoos J.S., Bond C., Rabe M., Colbert S.H., King R., Sayre M., Heisler M. (2013). Barriers and facilitators to learning and performing cardiopulmonary resuscitation in neighborhoods with low bystander cardiopulmonary resuscitation prevalence and high rates of cardiac arrest in Columbus, OH. Circ. Cardiovasc. Qual. Outcomes.

[37] Uny I., Angus K., Duncan E., Dobbie F. (2023). Barriers and facilitators to delivering bystander cardiopulmonary resuscitation in deprived communities: A systematic review. Perspect. Public Health.

[38] Shams A., Raad M., Chams N., Chams S., Bachir R., El Sayed M.J. (2016). Community involvement in out of hospital cardiac arrest: A cross-sectional study assessing cardiopulmonary resuscitation awareness and barriers among the Lebanese youth. Medicine.

[39] Fazel M.F., Mohamad M.H.N., Sahar M.A., Juliana N., Abu I.F., Das S. (2022). Readiness of Bystander Cardiopulmonary Resuscitation (BCPR) during the COVID-19 Pandemic: A Review. Int. J. Environ. Res. Public Health.

[40] Woollard M., Smith A., Whitfield R., Chamberlain D., West R., Newcombe R., Clawson J. (2003). To blow or not to blow: A randomised controlled trial of compression-only and standard telephone CPR instructions in simulated cardiac arrest. Resuscitation.

[41] Nishiyama C., Iwami T., Kawamura T., Ando M., Yonemoto N., Hiraide A., Nonogi H. (2010). Quality of chest compressions during continuous CPR; comparison between chest compression-only CPR and conventional CPR. Resuscitation.

